# FDMB-YOLOv11: Traffic police command gesture recognition method under different lighting conditions

**DOI:** 10.1371/journal.pone.0357212

**Published:** 2026-08-28

**Authors:** Xiaoyu Zhang, Baohua Guo, Sigama Anthony, David Bassir, Yuandi Zhang

**Affiliations:** 1 School of Energy Science and Engineering, Henan Polytechnic University, Jiaozuo, Henan, China; 2 Jiaozuo Engineering Research Center of Road Traffic and Transportation, Henan Polytechnic University, Jiaozuo, Henan, China; 3 Faculty of Science, Engineering and Agriculture, University of Venda, Thohoyandou, Limpopo, South Africa; 4 Centre Borelli, UMR CNRS 9010, ENS Paris‑Saclay University, 91190 Gif‑sur‑Yvette, France; 5 Smart Structural Health Monitoring and Control Laboratory, DGUT-CNAM, Dongguan University of Technology, Dongguan, Guangdong, China; 6 Dongbei University of Finance and Economics, International Business College, Dalian, Liaoning, China; Nanjing Forestry University, CHINA

## Abstract

To address the challenges of false detection, insufficient feature representation, and limited real-time performance in image-based traffic police command gesture detection under different lighting conditions, this paper proposes a detection model named FDMB-YOLOv11 based on the YOLOv11n architecture. Firstly, the detection head was enhanced by introducing Frequency Adaptive Dilated Convolution (FADC), while the BiFormer attention mechanism was incorporated into the C2PSA module. Secondly, MobileNetV4 was adopted as the backbone network to reduce computational complexity. Finally, standard convolutions were replaced with Deformable Convolution to improve feature representation capability. Experimental results show that, based on the improvements described above, the precision, recall, and mean average precision (mAP@0.5) of the FDMB-YOLOv11 model under normal lighting conditions reach 97.91%, 93.32%, and 97.17% respectively, which are 23.1%, 13.63%, and 16.74% higher than those of the YOLOv11n model. The model’s parameter count is reduced from 2.58 million to 1.93 million, achieving lightweight optimization. Although the frame rate (FPS) is reduced to 65.789 fps, the model still achieves real-time inference performance and demonstrates potential for deployment on edge devices. The model outperforms target detection algorithms like SSD, Faster R-CNN, RTDETR, YOLOv12, and YOLOv13 by achieving the highest mAP@0.5. It also addresses the misclassification problem seen in the YOLOv11n model regarding left-turn and right-turn gestures while maintaining an optimal balance between accuracy and parameter count.

## Introduction

The development of autonomous driving technology depends on effective interaction between vehicles and humans, with traffic police gestures playing a vital guiding role in scenarios such as missing traffic signs and signal failures [1]. Real-time and accurate recognition of traffic police gestures can provide reliable visual inputs for traffic conflict warning systems developed in previous traffic safety research. [2] For autonomous driving systems, recognizing traffic police gestures requires overriding the conventional decision-making logic, which typically involves high-definition maps, traffic signals, and traffic rules, posing the highest-level challenges to the system’s perception and decision-making modules. It is not merely a technical problem but a complex systems engineering task that involves functional safety, expected functional safety, and human-machine interaction (HMI). However, lighting conditions in traffic scenarios are highly unpredictable, and the processing capacity of on-board hardware is limited [3]. An overly complex model will increase deployment difficulty, raise adaptation costs, and may also result in insufficient frame rate and response delay, failing to meet the requirements of real-time interaction. Therefore, developing a traffic police command gesture recognition method that offers high robustness, high accuracy, and low complexity under different lighting conditions is highly significant.

Chinese traffic police use eight command gestures, including “Stop”, “Go straight”, “Turn right”, “Turn left”, “Wait for the left turn”, “Slow down”, “Lane change”, and “Pull over”. Different posture frames within the same continuous action sequence are assigned to the same gesture category and used for single-image detection. In early gesture recognition research, scientists primarily depended on manual feature extraction methods and traditional classifiers. These methods typically extracted various hand features (e.g., shape, motion, contour, and texture features) from images or videos, and edge detection algorithms were frequently employed to obtain gesture edges for calculating parameters such as perimeter and area [4]. In recent years, owing to their high precision, rapid detection capabilities, and robust performance, deep learning-based image recognition techniques have been widely adopted in autonomous driving systems. K et al. [5] combined the ESRGAN super-resolution enhancement network with the Faster R-CNN object detection model, and the proposed method achieved significantly better performance than traditional models and the two individual models. This provides an effective solution for object detection in low-quality image scenarios. Li et al. [6] proposed the Strawberry R-CNN model, which improves the feature extraction and pooling operations of Faster R-CNN. This model achieves accurate recognition and counting of strawberries, outperforming mainstream models in performance. Zhu et al. [7] proposed an improved SSD model, achieving detection accuracies of 72.4% and 19.9% on two datasets, with a model parameter count of 3.86$\times 10^{6}$ and a detection speed of 84 FPS. Tang et al. [8] proposed the data-driven DDR-Net model based on RetinaNet, which is applied to small object detection tasks. Ayachi et al. [9] evaluated the performance of the YOLOv1 to YOLOv9 series models in real-time object detection. They found that YOLOv9 achieves the optimal balance between speed, accuracy, and model efficiency, with a mAP of 85.54%, a parameter count of 7.10$\times 10^{6}$, and an inference speed of 34 f/s. Meanwhile, they pointed out that early YOLO models suffer from challenges such as low accuracy in detecting small objects and a large model size.

YOLO was proposed by Redmon, J. et al. It is a neural network-based algorithm for object detection, which has undergone continuous model improvements and optimizations [10,11]. Yang et al. [12] proposed an improved YOLOv5 algorithm that delivers real and reliable environmental perception information of vulnerable road users for emergency braking in autonomous driving technology. By inferring the specific positions and label information of all targets only once, end-to-end training and prediction can be streamlined. Hermens et al. [13] pointed out that YOLOv8 can effectively handle low-resolution images, as well as transparent objects and shape-changing objects. Sun et al. [14] proposed the SDF-YOLO algorithm, which achieves a 4.1% improvement in precision, a 1.7% increase in F1-score, and a 3.1% rise in mAP compared with the original YOLOv9. This effectively addresses the detection challenges, such as vehicle occlusion and road submergence in flood scenarios. Meng et al. [15] proposed the YOLOv11-EGM model. In both the bounding box detection (Box) task and the mask segmentation (Mask) task, its mAP@0.50-0.95 has achieved a significant improvement compared to the original YOLOv11 model, demonstrating excellent segmentation accuracy and robustness in complex image scenarios.

However, extreme lighting conditions present several technical challenges, including challenges with hand segmentation, reduced feature stability due to lighting and skin color differences, and inherent trade-offs between hardware compatibility and model performance. These challenges limit the accuracy and applicability of traditional gesture recognition methods in real-world situations, making it challenging to meet practical requirements [16]. Cho et al. [17] pointed out that lighting can cause target morphological distortion, significantly degrading object detection performance. Li et al. [18] pointed out that low-light environments contain numerous small, dense, and occluded targets. This causes traditional object detection models to face challenges such as information loss, computational redundancy, inadequate capture of complex features, and poor generalization ability, which can lead to missed detections and false positives, making it difficult to meet the required detection accuracy. Li et al. [19] noted that uneven lighting can cause blurred boundaries between targets and backgrounds in images, weaken detailed features, and further worsen background adhesion and partial occlusion challenges, thus reducing the accuracy of target segmentation boundaries and the overall detection integrity results.

To address the above challenges, some scholars have proposed YOLO series algorithms, yet several challenges persist. Jia et al. [20] pointed out that in low-light environments, some YOLO series models struggle to accurately detect pavement defects, such as cracks and potholes. In normal light conditions, although they achieve a certain detection effect, some models have high hardware and technology requirements, making them unsuitable for resource-constrained environments. Gong et al. [21] proposed models such as YOLOv5s. Under sufficient lighting, some of these models are prone to redundant detection boxes and false positives. Under normal lighting, they exhibit insufficient accuracy in recognizing subtle behaviors. Under low lighting conditions, challenges such as reduced image contrast and increased noise become apparent. Li et al. [22] proposed the FE-YOLO model, which is prone to being significantly affected by a noticeable increase in image noise and severe loss of details under faint light conditions. Liu et al. [23] proposed the YOLO-FOD model. Under faint light conditions, the model suffers from insufficient feature extraction, primarily affected by a sharp increase in image noise and severe blurring of details. Xiao et al. [24] proposed the DHSW-YOLO model, which can adapt to object detection under bright and dark lighting conditions. However, it exhibits insufficient adaptability to scenarios with gradual changes in lighting. In summary, existing YOLO series models and their improved variants generally face challenges posed by lighting variations in object detection tasks. Under low-light conditions, they are prone to insufficient detection accuracy due to the effects of noise and detail loss. Even under normal or sufficient lighting, challenges such as redundant detections and poor recognition of subtle targets persist. Furthermore, some models have limited adaptability to scenarios with gradual lighting changes and high hardware requirements, making it difficult to fully meet the detection needs in complex lighting conditions.

Therefore, to address the challenges posed by traffic police command gesture recognition under varying lighting conditions, this study proposes the FDMB-YOLOv11 algorithm for recognizing the command gestures of Chinese traffic police, aiming to overcome the inherent limitations of existing methods. In this study, the detection head of the original model is replaced with a Frequency Adaptive Dilated Convolution (FADC), which enhances the ability to capture high-frequency details and expands the receptive field, thereby enabling better understanding of the subtle differences between different command gestures. MobileNetV4 was incorporated into the backbone network. While maintaining detection accuracy, this significantly enhances the model’s computational efficiency, reduces the number of parameters and inference latency, and achieves model lightweighting. The BiFormer attention mechanism is integrated into the C2PSA module. Its characteristics, including the ability to simultaneously capture local details and global semantics, effectively fuse spatial positional and channel semantic information, and adopt grouped sparsity, enhance YOLOv11n’s capability to capture and screen key features of various gestures under different lighting conditions. Furthermore, the standard convolution is replaced with Deformable Convolution (DConv). The main contradiction between “fixed rectangular receptive fields and complex target morphologies” is addressed through “dynamic receptive fields,” which significantly enhances the detection accuracy of irregular, occluded, and pose-variable targets while maintaining model lightweighting.

Through these innovations, the proposed method not only addresses the limitations of existing approaches, such as inadequate feature extraction, compromised real-time detection, and excessive model complexity under different lighting conditions, but also further improves the model’s detection accuracy, thereby providing reliable gesture command recognition support for intelligent transportation scenarios.

## Methodology and principles

### The YOLOv11n model

**Fig 1:**
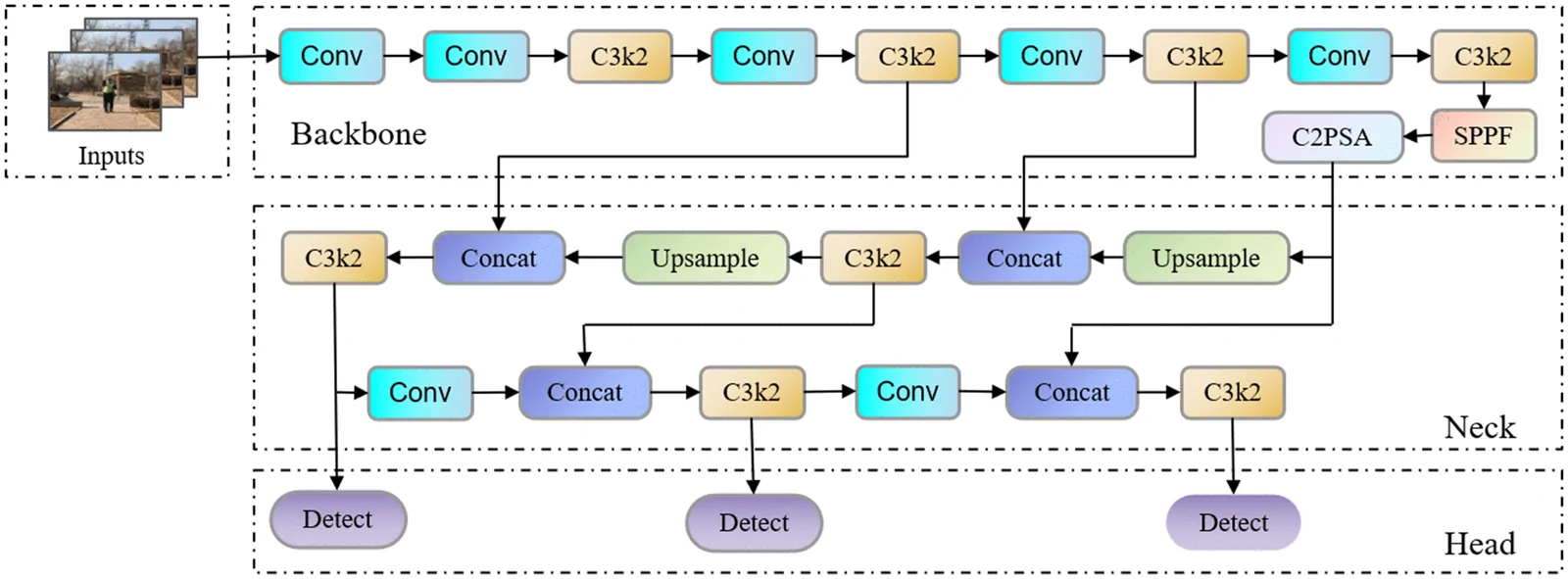
Schematic diagram of the YOLOv11n architecture. (Reprinted from Chinese Traffic Police Command Gesture Dataset (CC BY 4.0, copyright 2019), https://github.com/zc402/ChineseTrafficPolicePose.).

The YOLOv11 model integrates state-of-the-art technical modules, including C3k2 blocks, Spatial Pyramid Pooling Fusion (SPPF), and Channel and Positional Spatial Attention (C2PSA). These components significantly enhance its feature extraction capability and improve the recognition accuracy of multi-scale targets [25]. Compared with previous versions (e.g., YOLOv7, YOLOv8, YOLOv9, and YOLOv10), the YOLOv11 model exhibits superior feature extraction capability, higher training efficiency, broader task adaptability, and enhanced compatibility with edge computing [26]. In this study, the YOLOv11n model is selected for training on the dataset, and its structural schematic diagram is illustrated in Fig 1. The weights corresponding to the minimum loss on the validation set are used as the final weights, with a threshold of 0.5. Tests are conducted under normal lighting conditions, and the recognition results of each command gesture are presented in Table 1.

**Table 1:** Recognition results of the YOLOv11n model on the test set.

Category	P	R	F1	mAP@0.5
Lane change	0.7505	0.9000	0.8185	0.916
Go straight	0.9297	0.9259	0.9278	0.961
Prepare	0.8692	1.0000	0.9300	0.985
Pull over	0.9609	1.0000	0.9801	0.995
Slow down	1.0000	0.9333	0.9655	0.995
Stop	0.8672	0.9231	0.8943	0.916
Turn left	0.0000	0.0000	0.0000	0.083
Turn right	0.4114	0.7531	0.5321	0.456
Wait for the left turn	0.9443	0.7370	0.8278	0.932
Overall Evaluation Value	0.7481	0.7969	0.7718	0.804

As shown in Table 1, the YOLOv11n model’s recognition performance for left-turn and right-turn gestures exhibits significant discrepancies compared to other gestures. Specifically, the Precision, Recall, and F1-score for left-turn gesture recognition are all 0, with the mean Average Precision at IoU = 0.5 (mAP@0.5) merely 0.083; the right-turn gesture yields recognition results: its Precision is 0.4114, Recall is 0.7531, F1-score is 0.5321, and mAP@0.5 is 0.456, all of which are relatively low. The root cause of this challenge lies in the YOLOv11n model’s consistent misclassification of both left-turn and right-turn gestures as right-turn gestures, which leads to gesture misjudgment and directly results in low recognition accuracy for both gesture categories.

YOLOv11 employs a dual-prediction head architecture, comprising bounding box regression and class prediction tasks. Specifically, the classification head branch employs a lightweight structure of “depthwise separable convolution + 1×1 convolution,” which reduces the computational complexity by over 50% while maintaining comparable classification accuracy. The neck employs a simplified Path Aggregation Network (PAN) structure, with fewer fusion layers than large-scale models, and the number of feature channels per layer is less than 50% that of YOLOv11s. However, the feature maps output by the neck network still rely on traditional convolution operations, which leads to significant limitations in modelling local feature correlations [27]. The backbone network is the core for the model to extract basic features. In traffic police gesture recognition tasks, ordinary convolutions struggle to capture the subtle differences between left-turn and right-turn gestures, resulting in ambiguity in determining the direction of left and right turns. Therefore, this study will enhance four components of YOLOv11n: the convolutional layers in the detection head, backbone, and neck, as well as the C2PSA module. It will also conduct ablation experiments to determine the optimal improvement scheme.

### Improved detection head

**Fig 2:**
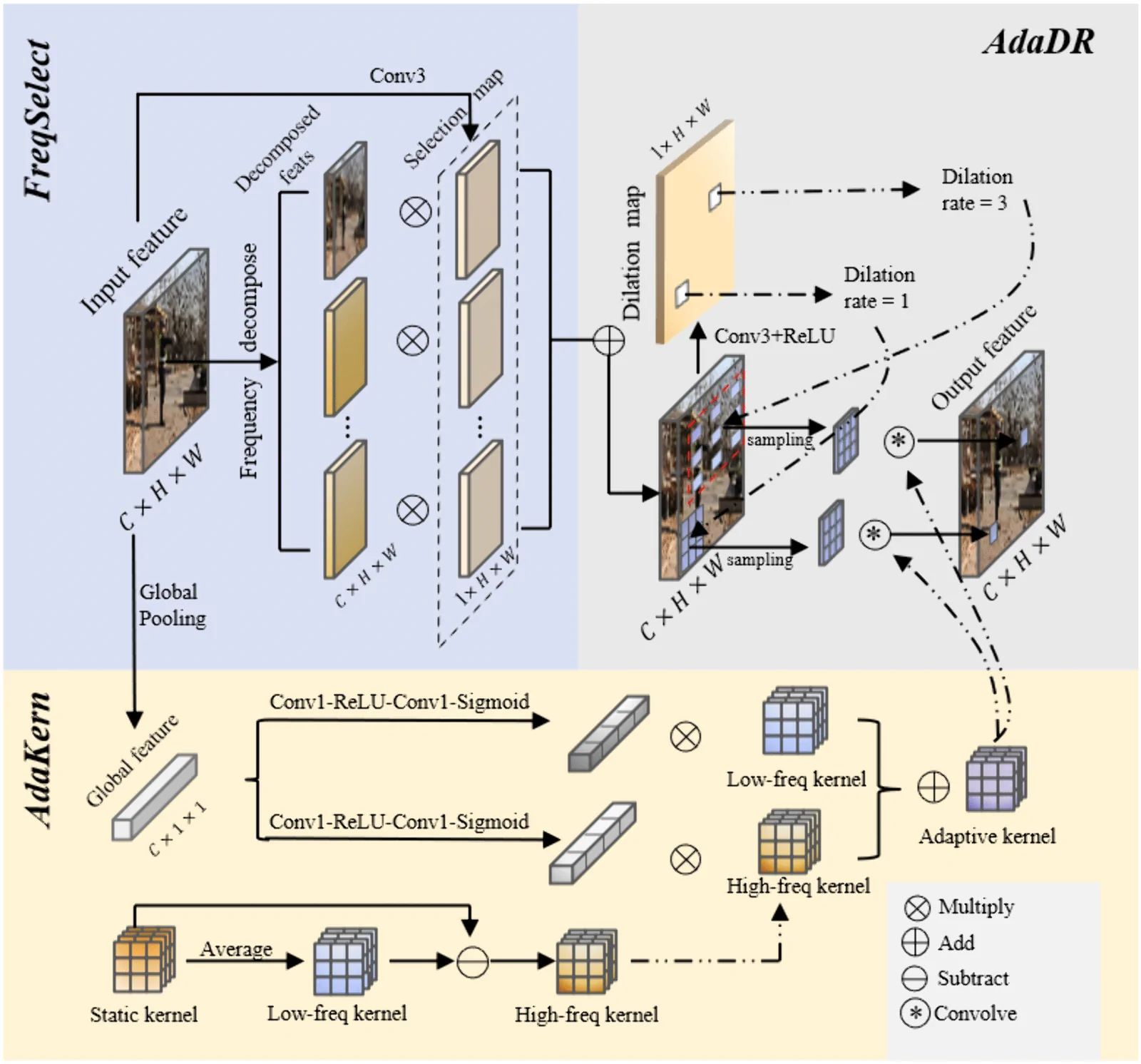
Network structure of FADC. (Reprinted from Chinese Traffic Police Command Gesture Dataset (CC BY 4.0, copyright 2019), https://github.com/zc402/ChineseTrafficPolicePose.).

The detection head of YOLOv11n is directly responsible for the final classification and localization of features. Therefore, this study introduces the FADC to improve the detection head of YOLOv11n. FADC enhances dilated convolution from a frequency analysis perspective, including three key strategies: Adaptive Dilation Rate (AdaDR), Adaptive Kernel (AdaKern), and Frequency Selection (FreqSelect). Specifically, AdaDR dynamically adjusts the dilation rate based on local frequency spectra, AdaKern optimizes the weights of convolution kernels, and FreqSelect directly balances the frequency power of input features, all designed to effectively expand the receptive field [28]. The integration of the FADC into the detection head enables the network to dynamically adapt its receptive field based on local variations in image content. This helps the model to better capture detailed image features in scenarios such as traffic police command gestures, thereby enhancing overall performance. The network structure of the FADC is shown in Fig 2.

#### Adaptive dilation rate (AdaDR).

The choice of dilation rate involves balancing large receptive fields with effective bandwidth. AdaDR assigns different dilation rates to each pixel, allowing a better trade-off between the spatial variations in input feature maps and the optimal dilation for each pixel. The dilated convolution used in AdaDR is described by Eq (1).


$$\begin{array}{c} Y(p)=\sum_{i=1}^{K\times K}W_{i}X\left(p+\Delta p_{i}\times \hat{D}(p)\right) \end{array}$$ (1)

Herein, *Y*(*p*) denotes the pixel value at position *p* in the output feature map, *K* represents the convolution kernel size, $W_{i}$ denotes the weight parameter of the convolution kernel, and $X(p+\Delta p_{i})$ denotes the pixel value at the corresponding position in the input feature map that is offset by $\Delta p_{i}$ relative to position *p*. The variable $\Delta p_{i}$ represents the *i*-th position of the predefined grid sampling, and its values include (-1,−1), (-1, 0), (−1,+1), ・・・, (+1, + 1) (i.e., when the convolution kernel covers the input feature map, the coordinate offset of each kernel element relative to the central position *p*; for a 3×3 convolution kernel includes 9 such offset positions).

Compared to the fixed dilation rate *D* in traditional dilated convolution, the dilation rate $\hat{D}(p)$ employed by AdaDR is predicted via a convolutional layer with parameter θ, aiming to maximize the receptive field while minimizing the frequency information loss for each pixel. Increasing the dilation rate $\hat{D}(p)$, expands the receptive field. Since the dilation rate cannot be negative, a ReLU layer is used to enforce non-negativity, and combined with the “modulation mechanism,” the dilation rate is adapted to the feature requirements.

*X*^(*p*,*s*)^ is a local feature centered at *p* with a window size of *s*, and its receptive field $RF(p)=(K-1)\times \hat{D}(p)+1$ is positively correlated with $\hat{D}(p)$. When the dilation rate increases, some high-frequency information will be lost, and the high-frequency power *HP*(*p*) is used to quantify the degree of loss, where $HP(p)=\sum_{{ℋ}_{\hat{D}(p)}^{+}}{\left|X_{F}^{\left(p,s\right)}(u,v)\right|}^{2}$, and ${ℋ}_{\hat{D}(p)}^{+}$ is the set of high-frequency regions. Therefore, to balance “receptive field expansion” and “high-frequency information loss”, a loss function is designed to optimize θ, as shown in Eq (2).


$$\begin{array}{c} \theta =\max_{\theta }\left(\sum RF(p)-\sum HP(p)\right) \end{array}$$ (2)

#### Adaptive kernel (AdaKern).

Traditional convolution kernels are fixed after training, whereas AdaDR can optimise their dynamic properties. To adapt the high-frequency and low-frequency components of convolution kernels in real-time, AdaKern is introduced to enable frequency adaptivity through decomposition and weighting. The decomposition of the static convolution kernel *W* is illustrated in Eq (3). After decomposition, the convolution kernel weights are recombined using low-frequency dynamic weights and high-frequency dynamic weights, as shown in Eq (4).


$$\begin{array}{c} W=\overset{―}{W}+\hat{W} \end{array}$$ (3)

$$\begin{array}{c} W^{\prime }=\lambda_{l}\overset{―}{W}+\lambda_{h}\hat{W} \end{array}$$ (4)

Herein, $\overset{―}{W}$ represents the kernel-level mean, responsible for maintaining low-frequency and global features, while $\overset{―}{W}=\frac{1}{K\times K}\sum_{i=1}^{K\times K}W_{i}$. $\hat{W}$ is the residual component that captures high-frequency and local details. $\hat{W}=W-\overset{―}{W}$ is the ratio $\frac{\lambda_{l}}{\lambda_{h}}$, which is dynamically adjusted based on the input context, allowing the network to focus on specific frequency bands and adapt to the complexity of visual patterns in features. This improves the network’s ability to capture low-frequency context and high-frequency local details, thereby increasing its overall effectiveness bandwidth.

#### Frequency selection (FreqSelect).

Traditional convolution functions as a high-pass filter, often showing a higher proportion of high-frequency components, which reduces the receptive field. FreqSelect can balance high-frequency and low-frequency components in feature representation, thereby improving the receptive field [29]. First, FreqSelect uses the Inverse Fast Fourier Transform ${ℱ}^{-1}$, combined with a binary mask, to decompose features into different frequency bands, as shown in Eq (5). Dynamic weighting is applied to the decomposed frequency bands, as shown in Eq (6).


$$\begin{array}{c} X_{b}={ℱ}^{-1}\left({ℳ}_{b}X_{F}\right) \end{array}$$ (5)

$$\begin{array}{c} \hat{X}(i,j)=\sum_{b=0}^{B-1}A_{b}(i,j)X_{b}(i,j) \end{array}$$ (6)

Herein, ${ℳ}_{b}(u,v)=\left\{ \begin{array}{cc} 1 & \text{if }\phi_{b}\leq \max(|u|,|v|)<\phi_{b+1}\\ 0 & \text{otherwise} \end{array}\right.\;$, where $\phi_{b}$ and $\phi_{b+1}$ are from B + 1 predefined frequency thresholds $\left\{0,\phi_{1},\phi_{2},...,\phi_{B-1},\frac{1}{2}\right\}\;$, $\hat{X}(i,j)\;$ is the frequency-balanced feature learned after FreqSelect, and $A_{b}\in {\mathbb{R}}^{H\times W}$ denotes the selection map of the *b*-th frequency band.

### Backbone improvement

MobileNetV4 is incorporated into the backbone of YOLOv11n. Through innovative architectural designs and optimization techniques, an efficient neural network capable of achieving real-time interaction performance is developed, breaking through the bottleneck of resource-constrained scenarios. The core building block of MobileNetV4, the Universal Inverted Bottleneck (UIB), integrates the Inverted Bottleneck (IB), ConvNext, Feed-Forward Network (FFN), and a novel Extra Depthwise (ExtraDW) variant into a unified and flexible structure. These four variants exhibit a significant trade-off among network depth expansion, receptive field control, and computational efficiency [30,31].

**Fig 3:**
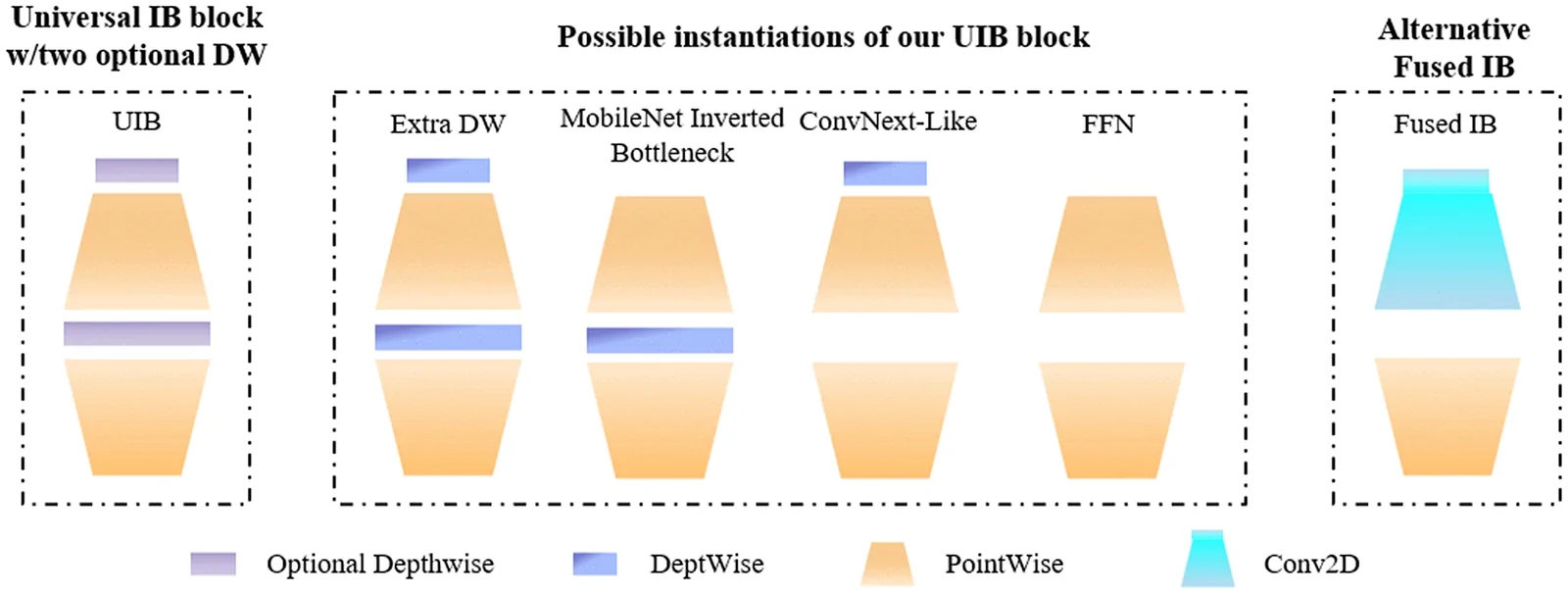
Universal Inverted Bottleneck (UIB) Module.

Fig 3 illustrates the structural diagram of the MobileNetV4 building block, known as the Universal Inverted Bottleneck (UIB) Module. When MobileNetV4 is used, the input image passes through a ConvBN module for downsampling. This module captures low-level gesture features, such as edge contours, local textures, and overall shapes, using small convolution kernels (e.g., 3×3). By applying a convolution with a stride of 2, the image’s spatial dimensions are decreased, reducing unnecessary computations and filtering out irrelevant background information, thereby improving the relative expression intensity of gesture features.

Meanwhile, the UIB module can flexibly adapt to the optional Depthwise Separable Convolution (DSC) according to scenarios. When the background is complex, DSC is enabled to reduce computation while enhancing the ability to capture local details. When the scenario is simple, DSC is disabled to improve inference speed, thereby balancing lightweight performance and accuracy. Furthermore, after passing through the UIB module, the spatial resolution of gesture images is further reduced. However, the receptive field is expanded through the stacking of multiple convolution layers, which can cover the entire gesture region and ensure the model’s understanding of the overall posture of “arm + palm.” Therefore, when MobileNetV4 is selected as the backbone network, it can output hierarchical features with different spatial resolutions, enabling rich semantic extraction and achieving model lightness.

### Improved convolutions

**Fig 4:**
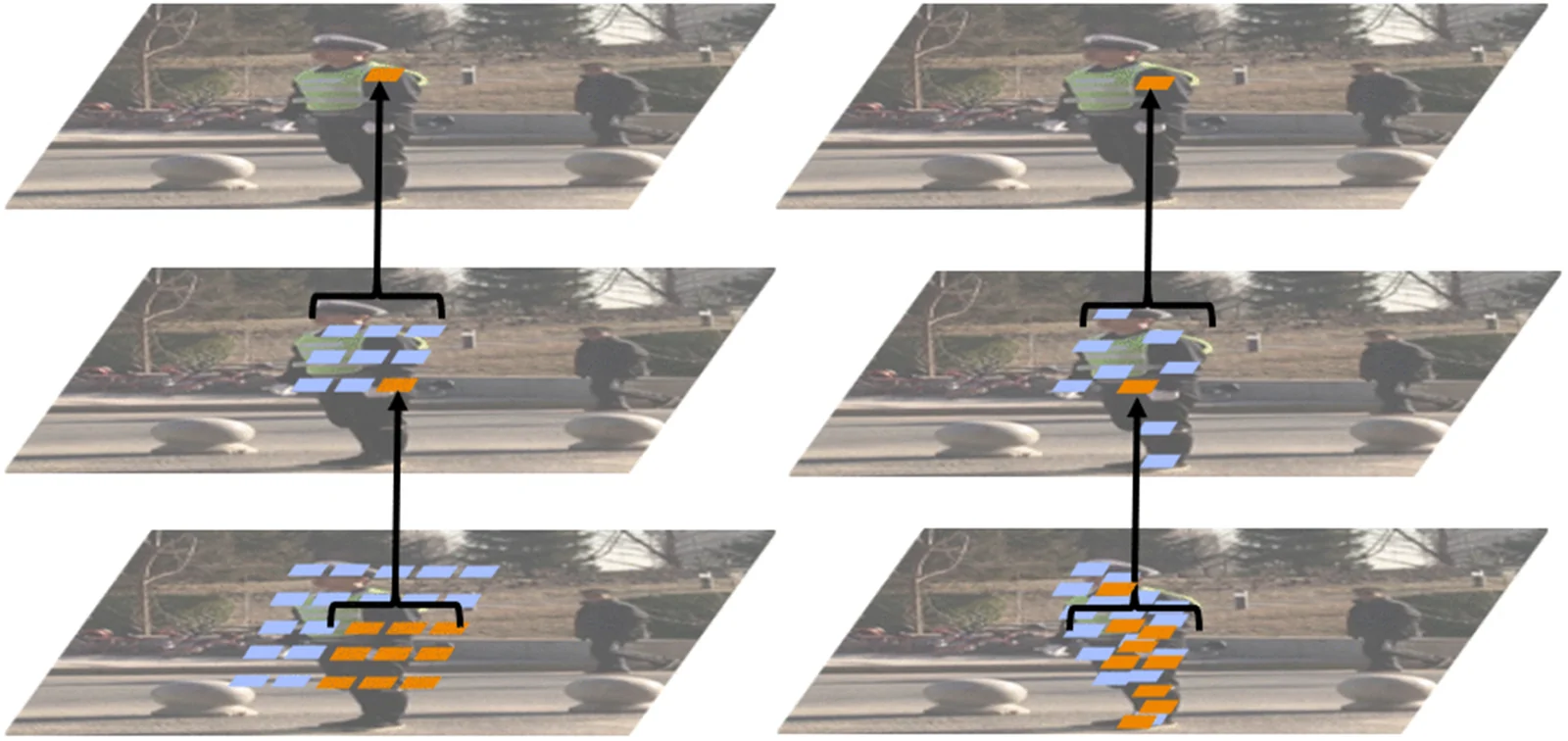
Receptive fields and sampling positions of standard convolution and deformable convolution. The standard convolution (left). The deformable convolution (right). (Reprinted from Chinese Traffic Police Command Gesture Dataset (CC BY 4.0, copyright 2019), https://github.com/zc402/ChineseTrafficPolicePose.).

The standard convolutions in YOLOv11n are replaced with Modulated Deformable Convolutionv2 (ModulatedDeformConv). The core objective is to enhance the model’s detection robustness against irregular, non-rigid, and occluded targets in complex scenarios, thereby addressing the inherent limitation of standard convolutions in fixed grid sampling patterns. In standard convolutions, the receptive field and sampling positions are fixed in the feature map, whereas in Deformable Convolution, they are adaptively adjusted according to the scale and shape of objects in the image [32], as illustrated in Fig 4.

**Fig 5:**
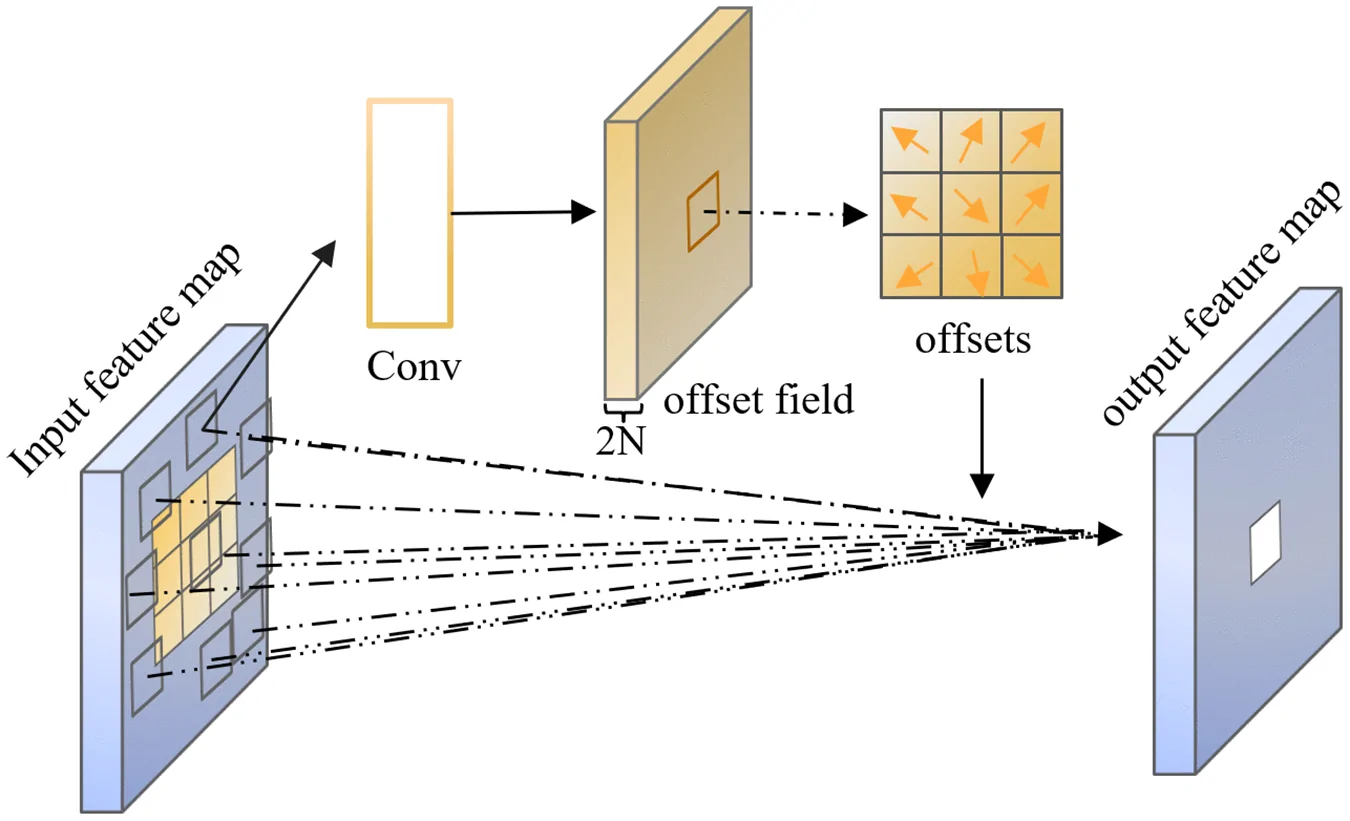
Structure of Deformable Convolution.

The principle of Deformable Convolution involves splitting the sampling process of standard convolution into two parallel branches that share the same input feature map. As shown in Fig 5, the upper branch uses an additional 1×1 convolution layer to compute the input feature map, ultimately producing an “offset matrix” with a shape of H×W×2N, where 2*N* indicates the number of offsets needed for all sampling points within a single convolution window. Based on the offsets generated by the upper branch, the lower branch shifts the original regular sampling window to new positions, completing the convolution computation.

The structure of Deformable Convolution is defined as shown in Eq (7).


$$\begin{array}{c} y(p_{0})=\sum_{p_{n}\in ℜ}w(p_{n})\cdot x\left(p_{0}+p_{n}+\Delta p_{n}\right) \end{array}$$ (7)

Herein, *y*(*p*_0_) denotes the value of the output feature map at position *p*_0_; $p_{n}$ is the relative sampling position within the receptive field ℜ of the convolution kernel; $w(p_{n})$ is the convolution weight at the corresponding position; $x\left(p_{0}+p_{n}+\Delta p_{n}\right)$ denotes the input feature map; and $\Delta p_{n}$ is the offset computed by the upper branch, which is used to adjust the position of each sampling point $p_{n}$.

Since the sampling positions become irregular, the offset $\Delta p_{n}$ is usually a fractional value. Therefore, it is implemented via bilinear interpolation, as shown in Eq (8).


$$\begin{array}{c} \begin{array}{c} \begin{array}{cc} x(p) & =\sum_{q}G(q,p)\times x(q)\\ & =\sum_{q}g\left(q_{x},p_{x}\right)\times g\left(q_{y},p_{y}\right)\times x(q)\\ & =\sum_{q}\max\left(0,1-|q_{x}-p_{x}|\right)\cdot \max\left(0,1-|q_{y}-p_{y}|\right)\cdot x(q) \end{array} \end{array} \end{array}$$ (8)

Herein, $p=p_{0}+p_{n}+\Delta p_{n}$ is the continuous sampling position after offset; *q* is the nearest integer coordinate point to *p* on the input feature map; *G*(*q*,*p*) is the weight of bilinear interpolation, which is used to compute the feature value *x*(*p*) at position *p* based on the feature values of surrounding integer coordinate points. This approach addresses the sampling challenge when the offset is a fractional value, ensuring the continuity and accuracy of feature extraction.

ModulatedDeformConv further introduces an additional modulation scalar mechanism based on the aforementioned Deformable Convolution, and its structural definition is extended as shown in Eq (9).


$$\begin{array}{c} y(p_{0})=\sum_{p_{n}\in ℜ}\Gamma (p_{n})\cdot w(p_{n})\cdot x\left(p_{0}+p_{n}+\Delta p_{n}\right) \end{array}$$ (9)

$\Gamma (p_{n})$ is the modulation scalar with a value range of [0, 1], used to weigh the contribution of each sampling point after offset. When the target is occluded, or local features are insignificant, $\Gamma (p_{n})$ can decrease the weight of the corresponding sampling points, enabling the model to concentrate more on key effective features and further improving the detection robustness of targets in complex scene scenarios.

### Improved C2PSA module

**Fig 6:**
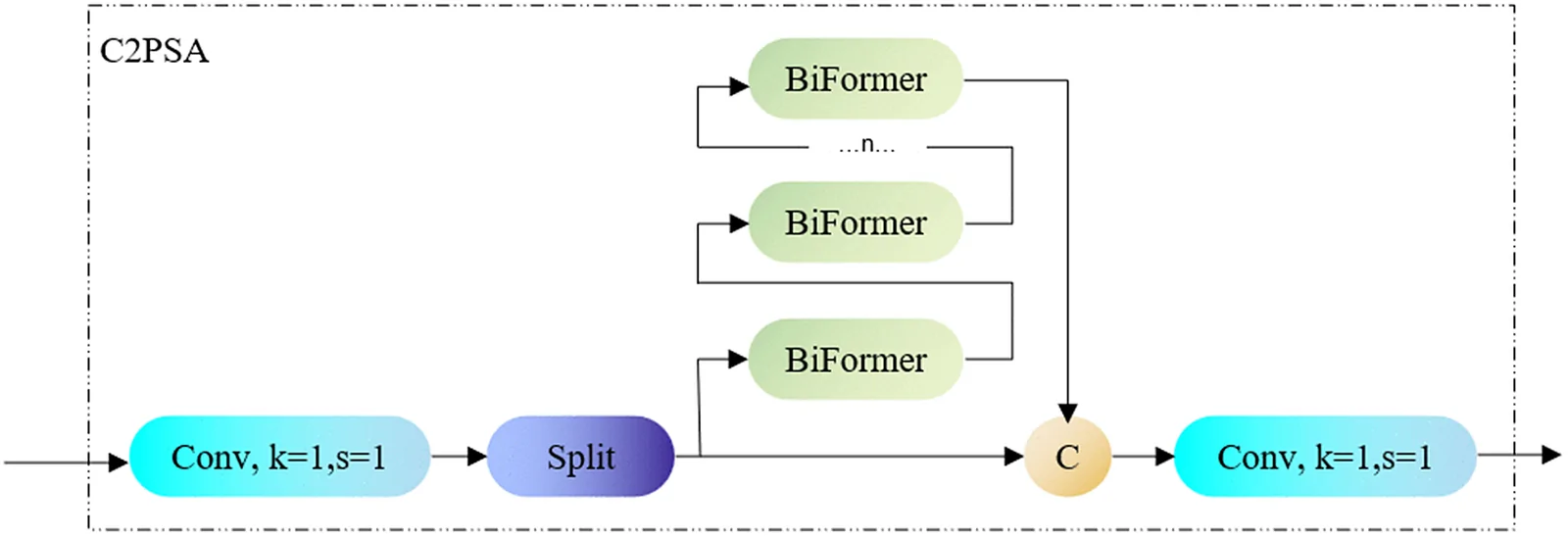
C2PSA mechanism optimized with the BiFormer attention mechanism.

In YOLOv11n, the core feature extraction component of the C2PSA attention mechanism is responsible for combining local and global information to improve the model’s ability to focus on important regions in images, allowing for more effective detection of small objects or partially occluded one’s objects. The position-sensitive attention (PSA) within C2PSA depends on fixed window partitioning. If the window fails to accurately capture the key regions of gestures, local detail features will be averaged out. In contrast, BiFormer precisely targets the critical parts of gestures through a dual-path focusing mechanism. Therefore, this paper introduces the BiFormer attention mechanism to enhance the C2PSA mechanism in YOLOv11n, as illustrated in the structural diagram shown in Fig 6.

**Fig 7:**
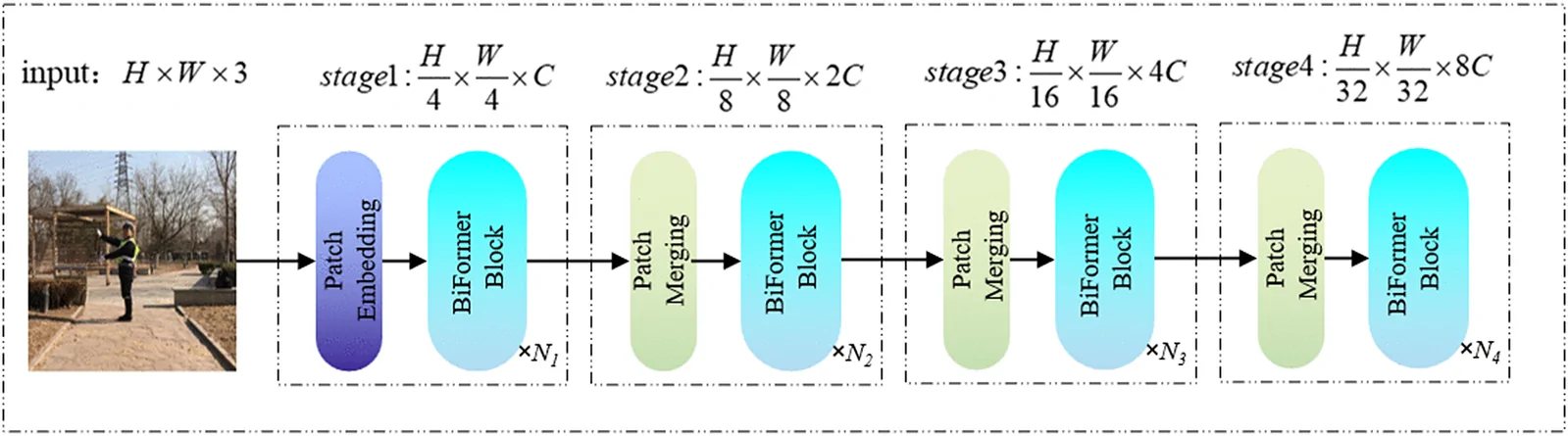
Schematic diagram of the BiFormer structure. (Reprinted from Chinese Traffic Police Command Gesture Dataset (CC BY 4.0, copyright 2019), https://github.com/zc402/ChineseTrafficPolicePose.).

BiFormer is a novel dynamic sparse attention mechanism that enables flexible allocation of computational resources through a dual-path structure. It focuses on a small subset of relevant tokens in a query-adaptive manner, thereby eliminating interference from other irrelevant tokens. Boasting excellent performance and high computational efficiency [33], its structural schematic is illustrated in Fig 7. The model processes RGB images of size H×W×3 in four sequential stages: In Stage 1, overlapping patch embedding is adopted to reduce the image resolution to $\frac{H}{4}\times \frac{W}{4}$ with the number of channels set to *C*; subsequently, feature extraction is performed via *N*_1_ serially connected BiFormer Blocks. In Stages 2–4, patch merging is utilized to further downscale the resolution to $\frac{H}{32}\times \frac{W}{32}$, while the number of channels is doubled incrementally to 8*C*.

The dataset used in this paper is sourced from a publicly available traffic police gesture dataset [34]. A total of 3,728 consecutive action frame images were collected from the public dataset at a sampling rate of 10 frames per second. An image enhancement method based on the HSV color space was employed to generate samples under different lighting conditions. Specifically, the brightness of images was controlled by adjusting the V channel, combined with contrast adjustment and noise perturbation to simulate real light imaging environments and cover light variations in practical traffic scenarios.

To adapt to real-world road lighting conditions, the illumination intensity is categorized into five levels: faint light (illumination intensity < 200 lux), low light (200 lux ⩽ illumination intensity < 500 lux), normal light (500 lux ⩽ illumination intensity < 2000 lux), strong light (2000 lux ⩽ illumination intensity < 5000 lux), and excessive light (illumination intensity ≥ 5000 lux). Through lighting processing techniques, 300 images were generated for each of the four conditions: faint light, low light, strong light, and excessive light, as well as 2,528 images under normal light conditions.

The subsets under different lighting conditions were composed of independent image samples, and no identical original image was repeatedly included in multiple lighting-condition subsets after applying different lighting transformations. Therefore, each lighting-condition subset can independently reflect the gesture detection performance under the corresponding environmental conditions.

The eight traffic police command gestures have different decomposed sequential motions, so we label samples following the decomposed actions. The preparatory posture before the start of each gesture is labelled as “Prepare”. Consequently, the annotation categories are divided into nine types: Lane change, Go straight, Prepare, Pull over, Slow down, Stop, Turn left, Turn right, and Wait for the left turn. During the annotation process, integers 0–8 are used to represent the nine categories of label information listed above.

To ensure the reliability of the experimental results, the dataset was divided into training, validation, and testing sets according to a ratio of train: val: test = 8:1:1. The dataset division was performed based on independent image samples, with no overlap among the training, validation, and testing sets. Meanwhile, the data under different lighting conditions were divided using the same ratio to ensure the fairness of the model training and evaluation process.

### Experimental environment configuration

The experiments were conducted on the Windows 11 operating system with the following computer configuration: CPU: 13th Gen Intel® Core™ i7-13650HX @ 2.60 GHz, RAM: 16.0 GB, GPU: NVIDIA GeForce RTX 4060 Laptop GPU. The deep learning platform was built based on the Ultralytics 8.3.0 framework. The programming language was Python 3.12.9, and the deep learning framework was PyTorch 2.5.1. GPU acceleration was implemented using CUDA 12.0.

The source code and pretrained weights of the YOLOv11n network were obtained from the Ultralytics open-source repository. To ensure experimental reproducibility, the random seed was fixed at 0, and deterministic training mode was enabled. During training, the data augmentation strategies included HSV color space augmentation, horizontal flipping (probability = 0.5), translation (factor = 0.1), scaling (factor = 0.5), Mosaic augmentation (probability = 1.0), random erasing (probability = 0.4), and RandAugment. Other geometric augmentation operations were disabled.

During inference, the confidence threshold was set to 0.25. A class-aware non-maximum suppression (NMS) strategy was adopted, with the intersection over union (IoU) threshold set to 0.7. All comparison algorithms were trained using the same dataset split (train:val:test = 8:1:1), 200 training epochs, identical data augmentation strategies, and consistent evaluation settings to ensure fair comparisons.

## Experiments and results

All models proposed in the experiments were trained and validated on the dataset covering five lighting levels, with the weights corresponding to the minimum validation loss selected as the optimal weights of the model. To clearly present the core performance of the models, all subsequent experimental analyses are based on the normal lighting conditions as the benchmark, and the specific results of various evaluation metrics are systematically presented and reported.

### Model evaluation metrics

For the YOLO target detection algorithm, the key metrics used to evaluate detection performance include Precision, Recall, F1 Score, mean Average Precision (mAP@0.5), model parameter count (Params/M), and Frames Per Second (FPS). Their calculation formulas are presented in Eq (10) through (13).


$$\begin{array}{c} P=\frac{TP}{TP+FP} \end{array}$$ (10)

$$\begin{array}{c} R=\frac{TP}{TP+FN} \end{array}$$ (11)

$$\begin{array}{c} mAP=\frac{\sum_{i=1}^{N}AP_{i}}{N} \end{array}$$ (12)

$$\begin{array}{c} F1=\frac{2PR}{P+R} \end{array}$$ (13)

Where: *TP* denotes true positives (correctly detected positive samples), *FN* denotes false negatives (missed positive samples), and *N* denotes the number of sample categories. In this study, *N* = 9. The FPS values reported in this study were obtained using the experimental hardware platform specified in the Experimental Environment Configuration section.

### Comparative experiments on the C2PSA mechanism

To assess the effectiveness of the C2PSA-BiFormer module, comparative experiments were carried out under standard lighting conditions. Keeping the same experimental setup, the C2PSA attention mechanism of the baseline model YOLOv11n was enhanced by replacing the original C2PSA module with C2PSA-SENetV2, C2PSA-MSDA, C2PSA-BiFormer, and C2PSA-DAT, respectively. The experimental results are presented in Table 2.

**Table 2:** Comparison of experimental results of different C2PSA modules on the test set.

Model	P	R	mAP@0.5	Params (M)	FPS $\left(f\cdot s^{-1}\right)$	F1
YOLOv11n+C2PSA-SENetV2	0.6972	0.8189	0.8100	2.54	204.08	0.7531
YOLOv11n+C2PSA-MSDA	0.8885	0.8767	0.9182	2.60	181.82	0.8826
YOLOv11n+C2PSA-BiFormer	0.7867	0.8904	0.9259	2.60	303.03	0.8353
YOLOv11n+C2PSA-DAT	0.8167	0.8624	0.8599	2.61	181.82	0.8389
YOLOv11n	0.7481	0.7969	0.8043	2.58	312.50	0.7718

As shown in Table 2, the mAP@0.5 of the original YOLOv11n is only 0.8043. In contrast, after integrating the improved C2PSA series modules, the mAP@0.5 of all modified models increases, confirming the optimization effect of the enhanced C2PSA mechanism on detection accuracy. Among them, the mAP@0.5 of YOLOv11n+C2PSA-BiFormer reaches 0.9259, a 12.16% improvement over the baseline model. Additionally, its F1 score (0.8353) and Recall (0.8904) are also maintained at high levels, making it the most accurate among all variants. Meanwhile, its parameter count (2.60 M) is nearly identical to that of the original model (2.58 M), and its FPS (303.03 f/s) is only marginally lower than the original model (312.50 f/s). This represents a significant breakthrough in improving accuracy without sacrificing efficiency. Conversely, for other variants (e.g., C2PSA-SENetV2), the mAP@0.5 is 0.8100, a mere 0.57% increase with a limited optimization effect. This indicates that different C2PSA modules have varying adaptability, with the BiFormer structure being more compatible with the current detection task requirements. Thus, based on a careful consideration of multidimensional metrics such as detection accuracy and inference speed, the C2PSA-BiFormer module is the optimal choice among all comparative modules and is therefore adopted in subsequent experiments.

### Ablation experiments

To evaluate the effectiveness of each proposed module enhancement in image-based traffic police command gesture detection, ablation experiments were conducted under normal lighting conditions. The experimental results are shown in Table 3, “√“ indicates that the corresponding component is improved or incorporated in the model.

**Table 3:** Results of ablation experiments on the test set.

Model	Head	Backbone	Conv	C2PSA	P	R	mAP @0.5	Params (M)	FPS $\left(f\cdot s^{-1}\right)$	F1
1					0.7481	0.7969	0.8043	2.58	312.50	0.7718
2	√				0.9855	0.9250	0.9702	2.66	51.282	0.9543
3	√	√			0.8507	0.9108	0.9383	1.87	53.45	0.8798
4	√		√		0.7497	0.8833	0.8237	2.71	49.751	0.8110
5	√			√	0.7339	0.7624	0.8145	2.67	48.31	0.7479
6	√	√	√		0.7198	0.6922	0.7833	1.92	66.67	0.7057
7	√	√	√	√	0.9791	0.9332	0.9717	1.93	65.789	0.9556
8		√			0.7809	0.9004	0.8437	1.79	434.78	0.8364
9		√	√		0.5737	0.7554	0.7693	1.84	322.58	0.6521
10		√		√	0.8366	0.8889	0.8910	1.81	238.10	0.8620
11		√	√	√	0.8320	0.8663	0.8762	1.86	227.27	0.8488
12			√		0.7675	0.9009	0.8326	2.62	250	0.8289
13			√	√	0.9130	0.8311	0.8311	2.61	185.18	0.8702
14				√	0.7867	0.8904	0.9259	2.60	303.03	0.8353

From the perspective of several key detection metrics in Table 3, improving a single module means that Model 2 includes the FADC module. Its Precision, Recall, mAP@0.5, and F1 score are improved to 0.9855, 0.9250, 0.9702, and 0.9543, respectively, showing increases of 23.74%, 12.81%, 16.59%, and 18.25% compared to the baseline YOLOv11n model. However, the model’s parameter count increases to 2.66 million, and the frame rate drops sharply to 51.282 f/s. Model 4 adds the ModulatedDeformConv module, with its mAP@0.5 improved to 0.8237, a 1.94% increase over the baseline. But the parameter count increases to 2.71 million, and the frame rate decreases to 49.751 f/s. This suggests that while these two individual modules do improve detection performance to some extent, they also increase the model’s computational complexity, thereby impacting real-time processing performance.

Model 8 introduces the MobileNetV4 module. Compared to the baseline model, its mAP@0.5 improves by 4.07%, with only 1.79 M parameters and a frame rate of up to 434.78 f/s. This indicates that although the MobileNetV4 module offers a less significant increase in detection performance compared to the FADC module, it can effectively reduce model complexity. Based on YOLOv11n, Model 14 incorporates the C2PSA-BiFormer module. Its mAP@0.5 rises to 0.9259, a 12.16% increase over the baseline model. This demonstrates that the module helps enhance overall performance. Although the parameter counts increase to 2.6 million, the frame rate still reaches 303.03 frames per second.

For multi-module improvements, Model 3 combines the FADC and MobileNetV4 modules. Its mAP@0.5 reaches 0.9383, representing a 13.4% increase compared to the baseline model, with a parameter count of 1.87 million and a frame rate of 53.45 frames per second. Compared to single-module improvements, while ensuring a certain degree of enhancement in accuracy and Recall, the model maintains well-controlled parameter counts. This suggests that the combination of these two modules exhibits a certain degree of complementarity, striking a balance between performance improvement and model complexity. Model 5 combines the FADC and C2PSA-BiFormer modules, with a Precision of 0.7339, a Recall of 0.7624, an mAP@0.5 of 0.8145, and an F1 score of 0.7479. Compared to Model 2, some metrics have conversely decreased after incorporating the C2PSA-BiFormer module. It is speculated that a certain degree of conflict exists between the two modules, failing to achieve a synergistic improvement effect. Model 10 incorporates the MobileNetV4 and C2PSA-BiFormer modules, achieving an mAP@0.5 of 0.891, representing an 8.67% improvement over the baseline model. The performance of this dual-module combination lies between the effects of individual single-module improvements, demonstrating a certain degree of synergistic impact; however, the magnitude of improvement is not particularly pronounced.

Model 12 combines the MobileNetV4 and ModulatedDeformConv modules, achieving a Precision of 0.7675, a Recall of 0.9009, an mAP@0.5 of 0.8326, a parameter count of 2.62 million, and a frame rate of 250.0 frames per second. Its performance also falls at a medium level, reflecting the diversity of the impacts of different module combinations on performance. Model 6 integrates the FADC, MobileNetV4, and ModulatedDeformConv modules, with a Precision of 0.7198, a Recall of 0.6922, an mAP@0.5 of 0.7833, and an F1 score of 0.7057. The performance of this combination is unsatisfactory, as all metrics are even lower than those of some single-module improvements and dual-module combinations. This indicates that an excessive number of module integrations may lead to performance degradation due to compatibility challenges. Model 7 integrates four modules (FADC, MobileNetV4, ModulatedDeformConv, and C2PSA-BiFormer). Its Precision, Recall, mAP@0.5, and F1 score are increased by 23.1%, 13.63%, 16.74%, and 18.38% respectively, compared to the baseline YOLOv11n model, with a parameter count of 1.93 M and a frame rate of 65.789 f/s. Model 7 achieves the best performance among all multi-module combinations and is named FDMB-YOLOv11. In summary, under an appropriate configuration, multiple modules can work synergistically to significantly enhance model performance. Although the frame rate has decreased to some extent, the overall detection effect remains excellent.

**Fig 8:**
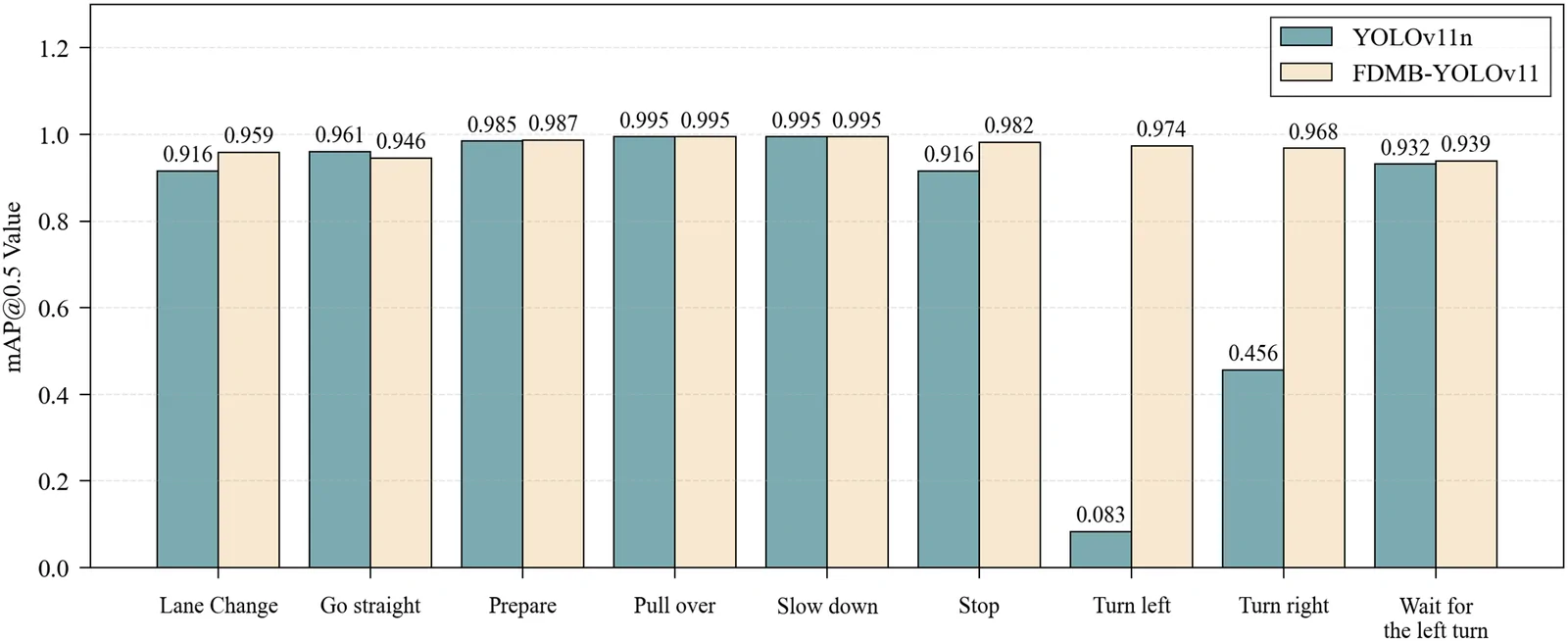
Visual comparison of mAP@0.5 performance between FDMB-YOLOv11 and YOLOv11n models on various gestures.

In summary, the model achieves optimal comprehensive performance when improvements are made simultaneously to its head, backbone, convolutional layers, and C2PSA module. The gesture recognition effects of the optimized model, FDMB-YOLOv11, and the baseline YOLOv11n are compared, as shown in Table 4 and Fig 8.

**Table 4:** Detection performance of FDMB-YOLOv11 and YOLOv11n for different traffic police command gestures on the test set.

Category	YOLOv11n				FDMB-YOLOv11			
	P	R	F1	mAP@0.5	P	R	F1	mAP@0.5
Lane Change	0.7505	0.9000	0.8185	0.916	0.9825	0.9500	0.9660	0.959
Go straight	0.9297	0.9259	0.9278	0.961	0.9949	0.9259	0.9592	0.946
Prepare	0.8692	1.0000	0.9300	0.985	0.9151	1.0000	0.9557	0.987
Pull over	0.9609	1.0000	0.9801	0.995	1.0000	0.9822	0.9910	0.995
Slow down	1.0000	0.9333	0.9655	0.995	0.9976	1.0000	0.9988	0.995
Stop	0.8672	0.9231	0.8943	0.916	0.9647	0.9231	0.9434	0.982
Turn left	0.0000	0.0000	0.0000	0.083	0.9682	0.9655	0.9669	0.974
Turn right	0.4114	0.7531	0.5321	0.456	1.0000	0.9128	0.9544	0.968
Wait for the left turn	0.9443	0.7370	0.8278	0.932	0.9891	0.7391	0.8460	0.939

**Fig 9:**
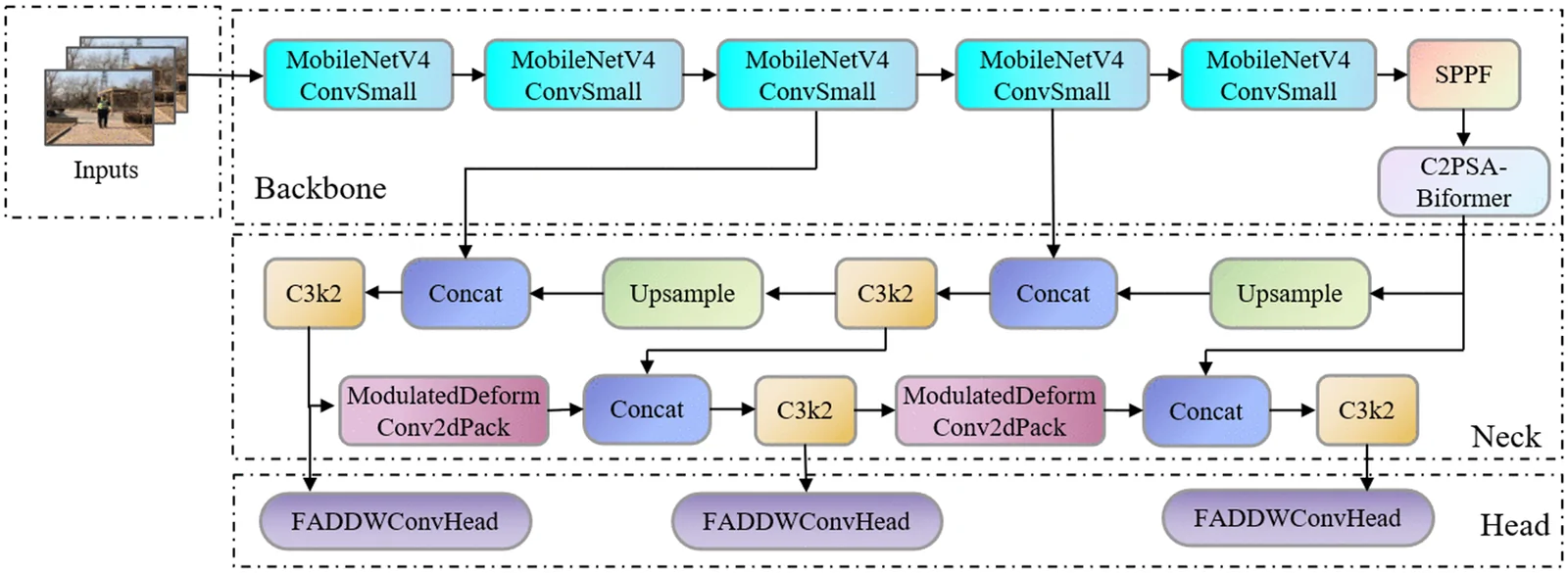
Network structure diagram of the FDMB-YOLOv11 model. (Reprinted from Chinese Traffic Police Command Gesture Dataset (CC BY 4.0, copyright 2019), https://github.com/zc402/ChineseTrafficPolicePose.).

As shown in Table 4 and Fig 8, the enhanced FDMB-YOLOv11 surpasses YOLOv11n comprehensively in recognizing various traffic police command gestures. Key metrics, including precision, F1 score, and mAP@0.5, all demonstrate significant improvements. FDMB-YOLOv11 achieves mAP@0.5 increases of 89.1% and 51.2% respectively, in the “Turn left” and “Turn right” gesture categories. For categories such as “Lane change” and “Stop,” the Precision and F1 scores have increased by over 10%, accompanied by a notable rise in mAP@0.5. Overall, FDMB-YOLOv11 demonstrates notable advantages in recognizing diverse traffic police command gestures. It resolves the gesture misjudgment issue of the YOLOv11n model and confirms the effectiveness of modifications to modules, including the model’s head, backbone, and convolution layers. The network structure diagram of the FDMB-YOLOv11 model is illustrated in Fig 9.

**Fig 10:**
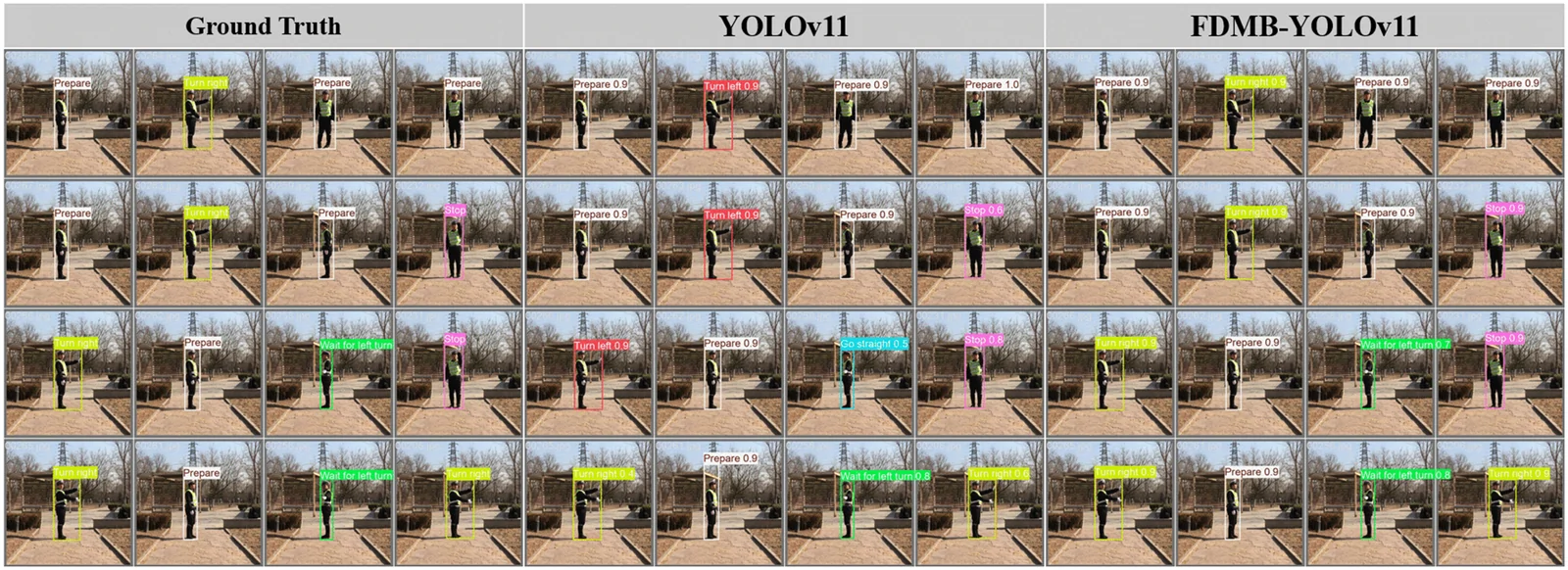
Comparison of detection results between YOLOv11 and FDMB-YOLOv11. (Reprinted from Chinese Traffic Police Command Gesture Dataset (CC BY 4.0, copyright 2019), https://github.com/zc402/ChineseTrafficPolicePose.).

As shown in Fig 10, a visual comparison of the typical detection results obtained by YOLOv11 and FDMB-YOLOv11 was conducted. The YOLOv11 model exhibited category confusion in several gestures with similar visual appearances, including left-turn versus right-turn gestures and left-turn waiting versus straight-going gestures, resulting in incorrect classification. In contrast, FDMB-YOLOv11 achieved more accurate discrimination among different gesture categories and effectively reduced false detections caused by similar postures.

### Comparative experiments

As shown in Table 5, when comparing the FDMB-YOLOv11 model with the traditional SSD and Faster R-CNN models, it is observed that the mAP@0.5 of FDMB-YOLOv11 increases by 6.1% and 1.6%, respectively. Not only does this model achieve the highest mAP@0.5, but it also boasts the highest frame rate (65.8 f/s > 40.38 f/s > 5.07 f/s) and the smallest parameter count (1.93 M < 2.48 M < 13.6 M). Compared with the newer YOLOv12 model, the mAP@0.5 of the FDMB-YOLOv11 model is 27% higher than that of YOLOv12, with a smaller parameter count (1.93 M < 2.51 M) and a slightly lower frame rate (65.8 f/s < 114.94 f/s). Compared with the existing traffic police command gesture recognition models YOLOv8n-Vanillanet-CARAFE-Triplet Attention and YOLOX-tiny, FDMB-YOLOv11 achieves the highest mAP@0.5 (0.971 > 0.965 > 0.883), the smallest parameter count (1.93 M < 2.13 M < 5.04 M), and a medium frame rate (55.21 f/s < 65.8 f/s < 81.8 f/s). Therefore, the FDMB-YOLOv11 model demonstrates superior overall performance in the image-based traffic police command gesture detection task, significantly outperforming traditional detection models and existing models designed for the same scenario.

**Table 5:** Performance comparison of different detection models on the test set.

Model	mAP@0.5	Params (M)	FPS $\left(f\cdot s^{-1}\right)$
FDMB-YOLOv11	0.972	1.93	65.8
YOLOv8n-Vanillanet-CARAFE-Triplet Attention [8]	0.965	2.13	81.8
YOLOX-tiny [35]	0.883	5.04	55.21
SSD	0.911	2.48	40.38
Faster-R-CNN	0.956	13.6	5.07
YOLOv12	0.701	2.51	114.94
YOLOv13	0.693	2.50	138.9
RTDETR	0.791	32.0	38.17

### Performance verification of the FDMB-YOLOv11 model under different lighting conditions

**Fig 11:**
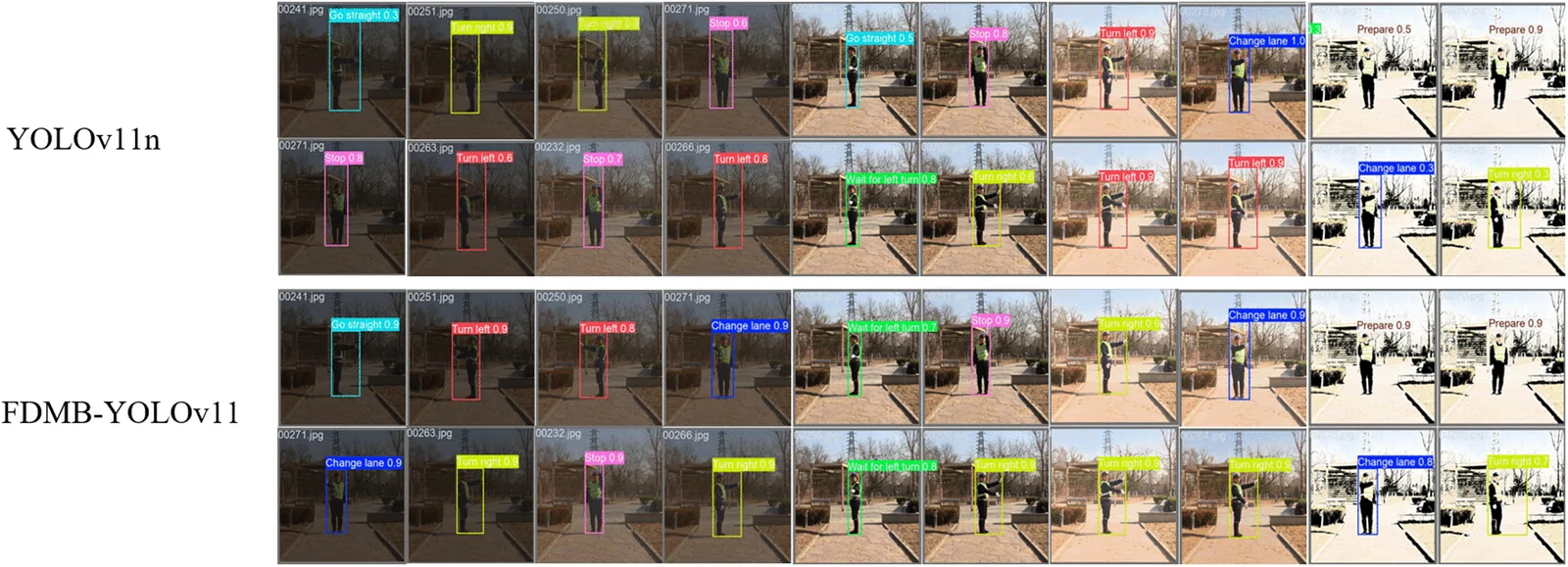
Recognition effect diagrams of the YOLOv11n model and the FDMB-YOLOv11 model under different lighting conditions. (Reprinted from Chinese Traffic Police Command Gesture Dataset (CC BY 4.0, copyright 2019), https://github.com/zc402/ChineseTrafficPolicePose.).

To verify the detection performance of the FDMB-YOLOv11 model in image-based traffic police command gesture detection under different lighting conditions, 371 traffic police command gesture images were selected for each lighting scenario (faint light, low light, normal light, Strong light, and excessive light). The YOLOv11n model and the FDMB-YOLOv11 model were used for prediction, respectively, and the various performance metrics are presented in Table 6. The recognition results of the models are illustrated in Fig 11.

**Table 6:** Detection performance of YOLOv11n and FDMB-YOLOv11 under different lighting conditions on the test set.

Lighting conditions	YOLOv11n				FDMB-YOLOv11			
	P	R	F1	mAP@0.5	P	R	F1	mAP@0.5
Faint light	0.3773	0.4574	0.4693	0.4135	0.8429	0.8906	0.9244	0.8661
Low light	0.6192	0.4243	0.5253	0.5036	0.8888	0.8701	0.9437	0.8793
Normal light	0.7481	0.7969	0.8043	0.7718	0.9791	0.9332	0.9717	0.9556
Strong light	0.6221	0.6426	0.7004	0.6322	0.9778	0.9308	0.9743	0.9537
Excessive light	0.7237	0.7014	0.7044	0.7123	0.7744	0.7928	0.8667	0.7835

From the quantitative metrics in Table 6, the FDMB-YOLOv11 model significantly outperforms the original YOLOv11n model in four key metrics, namely Precision, Recall, mAP@0.5, and F1 score, across the five different lighting conditions: faint light, low light, normal light, strong light, and excessive light. In faint lighting conditions, the mAP@0.5, Precision, Recall, and F1 score of FDMB-YOLOv11 increase by 45.51%, 46.56%, 43.32%, and 45.26%, respectively, compared with those of the YOLOv11n model. In the most challenging excessive light conditions for recognition, the mAP@0.5 of FDMB-YOLOv11 still reaches 0.8667, representing a 16.23% improvement compared with that of the YOLOv11n model. This fully demonstrates that the improved model has stronger adaptability to different lighting conditions.

Fig 11 shows the recognition effect diagrams of the YOLOv11n model and the FDMB-YOLOv11 model under different lighting conditions. The YOLOv11n model misjudges gestures such as lane changes, turns left, and turns right in low-light conditions, resulting in a low recognition rate for various gestures. It also exhibits misjudgment of left and right turn gestures in dim, normal, strong, and excessive light conditions. As shown in Tables 7 and 8, when recognizing left turn gestures across these five lighting conditions, the mAP@0.5 of the YOLOv11n model is nearly 0, while that of the FDMB-YOLOv11 model remains above 0.94, representing an improvement of over 94%. For right turn gesture recognition, the YOLOv11n model achieves an mAP@0.5 of nearly 0 in low-light and faint light conditions, whereas the FDMB-YOLOv11 model reaches 0.629 and 0.79, corresponding to increases of 59.26% and 77.59% respectively. In normal, strong, and excessive light conditions, the mAP@0.5 of the FDMB-YOLOv11 model increases by 51.2%, 76.63%, and 58.3%, respectively, compared to that of the YOLOv11n model. This fully confirms that the FDMB-YOLOv11 model outperforms the YOLOv11n model under all lighting conditions and can address the drawbacks existing in the YOLOv11n model.

**Table 7:** Detection performance of YOLOv11n and FDMB-YOLOv11 for turn-left gestures under different lighting conditions on the test set.

Lighting conditions	YOLOv11n				FDMB-YOLOv11			
	P	R	F1	mAP@0.5	P	R	F1	mAP@0.5
Faint light	0.0000	0.0000	0.0109	0.0000	0.9495	0.7586	0.9480	0.8434
Low light	0.0000	0.0000	0.0098	0.0000	1.0000	0.7222	0.9670	0.8387
Normal light	0.0000	0.0000	0.0829	0.0000	0.9682	0.9655	0.9740	0.9669
Strong light	0.0000	0.0000	0.0000	0.0000	0.9682	0.9655	0.9800	0.9670
Excessive light	1.0000	0.0528	0.2240	0.0731	0.9918	0.9655	0.9660	0.9785

**Table 8:** Detection performance of YOLOv11n and FDMB-YOLOv11 for turn-right gestures under different lighting conditions on the test set.

Lighting conditions	YOLOv11n				FDMB-YOLOv11			
	P	R	F1	mAP@0.5	P	R	F1	mAP@0.5
Faint light	0.0000	0.0000	0.0364	0.0000	0.4327	0.8462	0.6290	0.5726
Low light	0.0000	0.0000	0.0141	0.0000	0.4962	0.8462	0.7900	0.6255
Normal light	0.4114	0.7531	0.4560	0.5321	1.0000	0.9128	0.9680	0.9544
Strong light	0.0000	0.0000	0.2080	0.0000	0.9909	0.9231	0.9743	0.9558
Excessive light	0.3059	0.8077	0.3920	0.4437	0.4211	0.8393	0.9750	0.5608

## Discussion

The FDMB-YOLOv11 model achieves a high mAP@0.5 and a low parameter count, demonstrating notable advantages in image-based traffic police command gesture detection. Traffic police command gesture detection. However, it still has certain limitations. Firstly, although the model’s frame rate reaches 65.789 frames per second, which can meet real-time requirements, its detection speed remains slower compared to the YOLOv11n model. Further optimization is required for large-scale, real-time deployment scenarios. It is essential to employ model compression techniques, such as model pruning, knowledge distillation, and binarization, to enhance the model’s processing speed, minimize memory consumption, and enable efficient operation on embedded system devices.

Secondly, When the model is deployed in real-world traffic scenarios with mixed traffic flows [36], it is not only affected by varying lighting conditions but also faces recognition challenges caused by adverse weather conditions, such as rain and fog.

To further evaluate the adaptability of the proposed model under complex environmental conditions, simulated rainy and foggy scenarios were generated from the original test images using image degradation augmentation methods. Foggy images were synthesized by overlaying noisy thin haze layers and enhancing human gesture details, while rainy images were generated by randomly simulating raindrop patterns and applying transparent blending with the original images.

Based on this, the generalization capability and practical reliability of the FDMB-YOLOv11 model are further discussed, and the experimental results are presented in Table 9. The mAP@0.5 of the FDMB-YOLOv11 model reaches 0.9573 and 0.9224 in rainy and foggy weather, respectively, representing a 38.39% and 56.05% improvement compared with the YOLOv11n model. Although rainy weather involves interference such as a dark background and raindrop occlusion, the model still maintains excellent recognition performance. In foggy weather, gesture features are blurred due to haze occlusion, resulting in a slight decrease in recognition accuracy compared to rainy weather; however, the model still achieves a high average detection accuracy. It can be concluded that the model exhibits strong robustness, can cover a broader range of application scenarios, and is capable of meeting the complex practical requirements of intelligent transportation scenarios.

**Table 9:** Detection performance of YOLOv11n and FDMB-YOLOv11 under different weather conditions on the test set.

Lighting conditions	YOLOv11n				FDMB-YOLOv11			
	P	R	F1	mAP@0.5	P	R	F1	mAP@0.5
Rainy Weather	0.4630	0.4972	0.5734	0.4795	0.9466	0.8614	0.9573	0.9020
Foggy Weather	0.3598	0.3032	0.3619	0.3291	0.7891	0.8554	0.9224	0.8209

It is essential to note that the dataset used in the model’s training phase does not include images of traffic police command gestures under rainy or foggy conditions. Therefore, it cannot be confirmed whether the model’s detection performance will be further improved if samples under such weather conditions are added to the training dataset. Based on this, future research should further expand the traffic police command gesture dataset to cover different weather environments, verify the model’s detection performance after incremental training through experiments, and provide more sufficient support for the practical application of the model.

Finally, the proposed FDMB-YOLOv11 model currently relies on RGB images. In this study, the model takes individual static RGB images as input and does not utilize any temporal information from video streams or model the temporal relationships between consecutive frames. Therefore, for future video-based detection tasks, lightweight temporal modeling can be incorporated into the existing network by integrating depthwise separable 3D convolutions into the backend of the backbone network for processing consecutive frame feature sequences, or by introducing a compact single-layer LSTM after the neck output features to extract temporal information. These approaches allow the model to capture dynamic changes among gesture frames while avoiding a significant increase in model complexity.

## Conclusion

(1) This study proposes the FDMB-YOLOv11 model for Chinese traffic police gesture recognition under different lighting conditions, achieving an optimal balance between accuracy and parameter count. The model comprises four innovative components: a detection head based on FADC, a C2PSA-BiFormer module that reduces computational complexity, a MobileNetV4 backbone enabling model lightweighting, and Deformable Convolution that enhances the model’s adaptability to geometric transformations. In normal light conditions, the Precision, Recall, mAP@0.5, and F1 score of the FDMB-YOLOv11 model increase by 23.1%, 13.63%, 16.74%, and 18.38%, respectively, compared with the YOLOv11n model. Additionally, the model’s parameter count is reduced from 2.58 M to 1.93 M, demonstrating excellent performance.
(2) Ablation and comparative experiments were carried out under normal light conditions. The ablation experiments demonstrate that each module makes a significant contribution to the model’s final performance, underscoring its robustness and efficiency. Comparative experiments indicate that the FDMB-YOLOv11 model achieves the best overall detection performance compared to SSD, Faster-RCNN, YOLOv12, and existing traffic police command gesture recognition models. For recognizing left and right turn gestures, the mAP@0.5 of the FDMB-YOLOv11 model increases by 89.1% and 51.2%, respectively, compared with the YOLOv11n model, thereby resolving the gesture misjudgment issue of the YOLOv11n model.
(3) Under five lighting conditions (faint light, low light, normal light, strong light, and excessive light), the YOLOv11n model and the FDMB-YOLOv11 model were used for detection and prediction, respectively. The results show that the mAP@0.5 of the improved FDMB-YOLOv11 model increases by 45.51%, 41.84%, 16.74%, 27.39%, and 16.23% across the five lighting conditions, respectively. When recognizing left turn gestures, the mAP@0.5 improvement exceeds 94% in all cases, and the mAP@0.5 improvements for right turn gesture recognition are 59.26%, 77.59%, 51.2%, 76.62%, and 58.3% respectively. These results fully confirm that the FDMB-YOLOv11 model outperforms the YOLOv11n model under all lighting conditions.
